# Supplementary material for: Identification of Freezing-Responsive microRNAs and Their Targets in Chinese Jujube by Small RNA and Degradome Sequencing
Source: Int J Mol Sci. 2026 Sep 4;27(17):7912. doi: 10.3390/ijms27177912 (PMC13566890; doi:10.3390/ijms27177912)
Supplement: Supplementary file 1 [file ijms-27-07912-s001.zip › Supplementary Material S2/novel_miR_149.pdf]

Provisional ID : novel_miR_149
Score total : 266
Score for star read(s) : 3.9
Score for read counts : 259.6
Score for mfe : 0.8
Score for randfold : 1.6
Score for cons. seed :
Total read count : 521
Mature read count : 491
Loop read count : 0
Star read count : 30

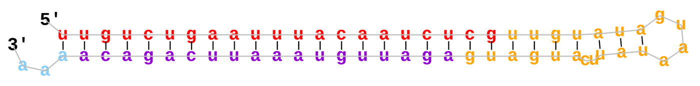

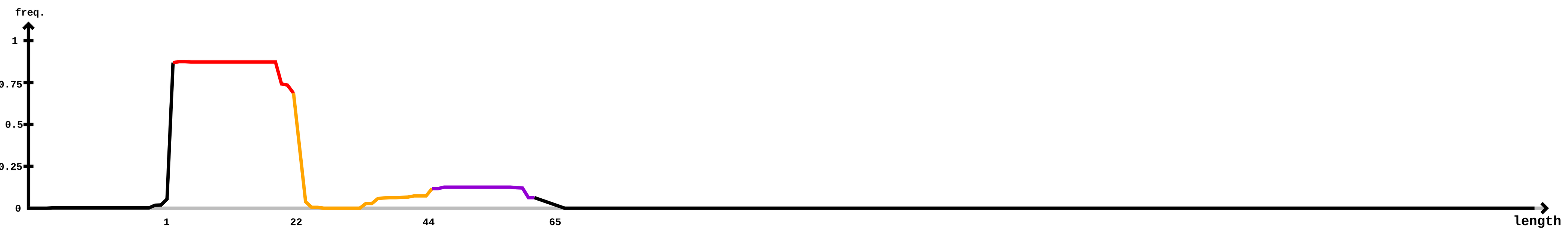

Mature Star

5'- uuaauuugcaagacauugaauuuugucugaauuuacaaucucguuguauaguaauauucaugaugagauuguaaauucagacaaaaugcaauucuuacaaaggucauuuuguuuguuugaugaauauagaguuauauauauauauauauauaaugaaauucuaugauaugaauaauacguaucauauuguaauauggauaucaauauugaugauuauuuuuaaaaaaauuaucguuaauauuuauuuu -3' obs

uuaauuugcaagacauugaauuuugucugaauuuacaaucucguuguauaguaauauucaugaugagauuguaaauucagacaaaaugcaauucuuacaaaggucauuuuguuuguuugaugaauauagaguuauauauauauauauauauauaaugaaauucuaugauaugaauaauacguaucauauuguaauauggauaucaauauugaugauuauuuuuaaaaaaauuaucguuaauauuuauuuu exp

.....((((((...((((.((((((((((((((((((((((((((((((((....)))..))))))))))))))))))))))))))))).))))((..(((((.(......).)))))..)).((((.....(((((((((((....)))))))))))...))))((((((((((.......)))))))))))))))...(((((..((((((((((((.(((((....))))).)))))))))))))))).	reads	mm	sample
..aauuugcaagacauugaauuuug	1	0	LT-1
...................auuuugucugaauuuacaauc	6	0	LCK-3
...................auuuugucugaauuuacaaucucg	2	0	JCK-2
...................auuuugucugaauuuacaaucucg	1	0	JCK-3
....................uuuugucugaauuuacaaucucguu	1	0	LT-3
.....................uuugucugaauuuacaaucuc	2	0	LCK-3
.....................uuugucugaauuuacaaucucguu	3	0	LT-3
.....................uuugucugaauuuacaaucucguu	2	0	JCK-3
.....................uuugucugaauuuacaaucucguu	4	0	LT-2
.....................uuugucugaauuuacaaucucguu	1	0	JCK-2
.....................uuugucugaauuuacaaucucguu	8	0	LT-1
......................uugucugaauuuacaauc	7	0	LT-3
......................uugucugaauuuacaauc	3	0	LT-2
......................uugucugaauuuacaauc	21	0	LCK-1
......................uugucugaauuuacaauc	3	0	LT-1
......................uugucugaauuuacaauc	27	0	LCK-2
......................uugucugaauuuacaCuc	1	1	LCK-3
......................uugucugaauuuacaauc	7	0	LCK-3
......................uugucugaauuuacaaucu	1	0	LT-1
......................uugucugaauuuacaauUu	2	1	LT-3
......................uugucugaauuuacaaucu	1	0	LT-3
......................uugucugaauuuacaauUuc	4	1	LT-2
......................uugucugCauuuacaaucuc	2	1	LT-1
......................uugucugaauuuacaaucuU	1	1	LT-1
......................uugucugaauuuacaaucuc	2	0	JCK-2
......................uugucugaauuuacaaucuc	2	0	LT-2
......................uugucugaauuuacaaucuc	8	0	LCK-3
......................uugucugaauuuacaaucuc	7	0	LT-1
......................uugucugaauuuacaaucucg	34	0	JCK-1
......................uugucugaauuuacaaucucg	53	0	LT-3
......................uugucugaauuuacaaucucg	64	0	LT-1
......................uugucugaauuuacaaucucU	17	1	LCK-2
......................uugucugaauuuaUaaucucg	1	1	JCK-3

Mature Star

uuaauuugcaagacauugaauuuugucugaauuuacaaucucguuguauaguaauauucaugaugagauuguaaauucagacaaaaugcaauucuuacaaaggucauuuuguuuguuugaugaauauagaguuauauauauauauauauauauaaugaaauucuaugauaugaauaauacguaucauauuguaauauggauaucaauauugaugauuauuuuaaaaaaauuaucguuaauauuuauuuu			
......................uugucugaauuuacaaucucg	51	0	JCK-2
......................uugucugaauuuacaaucucU	29	1	LCK-1
......................uugucugaauuuacaaucucg	12	0	LT-2
......................uugucugCauuuacaaucucg	1	1	LCK-3
......................uugucugaauuuacaaucucg	55	0	JCK-3
......................uugucugaauuuacaaucucg	50	0	LCK-3
......................uugucugaauuuacaaucucgA	1	1	LT-3
.......................ugucugaauuuacaaucucguugu	3	0	LT-1
....uauucaugaugagauuguaaauucaga	13	0	LCK-1
....uauucaugaugagauuguaaauucaga	3	0	LCK-2
....uucaugaugagauuguaaCuucaga	1	1	LCK-1
....uucaugaugagauuguaaauucaga	10	0	LCK-2
....uucaugaugagauuguaaauucaga	6	0	LCK-1
....ucaugaugagauuguaaauuca	2	0	LT-1
....caugaugagauuguaaauucagacU	1	1	LT-1
....ugaugagauuguaaauucag	1	0	LT-1
....gaugagauuguaaauucagaca	1	0	LT-1
....augagauuguaaauucagaca	2	0	JCK-1
....augagauuguaaauucagaca	2	0	LCK-3
....agauuguaaauucagaca	6	0	LT-2
....agauuguaaauucagaca	13	0	LCK-3
....agauuguaaauucagaca	1	0	JCK-3
....agauuguaaauucagacaaa	1	0	LT-1
....agauuguaaauucagacaaaa	3	0	LT-2
....agauuguaaauucagacaaaa	1	0	LT-1
....auuguaaauucagacaaaau	5	0	LCK-3
